# Effects of Concurrent High-Intensity and Strength Training on Muscle Power and Aerobic Performance in Young Soccer Players during the Pre-Season

**DOI:** 10.3390/sports11030059

**Published:** 2023-03-06

**Authors:** Pierros Thomakos, Konstantinos Spyrou, Christos Katsikas, Nikolaos D. Geladas, Gregory C. Bogdanis

**Affiliations:** 1 School of Physical Education and Sports Science, National and Kapodistrian University of Athens, 17237 Dafne, Greece; 2 UCAM Research Center for High Performance Sport, UCAM Universidad Catόlica de Murcia, 30005 Murcia, Spain; 3 Facultad de Deporte, UCAM Universidad Católica de Murcia, 30005 Murcia, Spain

**Keywords:** endurance, power, soccer, preseason, concurrent training

## Abstract

The aim of the present study was to evaluate two different intervention programs applied during a 4-week pre-season period. Twenty-nine players participated in this study and were divided into two groups. One group (Ball_Train_, *n* = 12, age: 17.8 ± 0.4 years, body mass: 73.9 ± 7.6 kg, height: 178 ± 0.1 cm, body fat: 9.6 ± 5.3%) performed a higher percentage of aerobic training with ball and strength training using plyometrics and exercises with body weight. The other group (HIIT_Train_, *n* = 17, age: 17.8 ± 0.7 years, body mass: 73.3 ± 5.0 kg, height: 179 ± 0.1 cm, body fat: 8.0 ± 2.3%) trained with high-intensity interval training (HIIT) without the ball and performed resistance training with weights in the same session. Both groups trained for strength (two times/week) and performed aerobic–anaerobic fitness without the ball, passing games, and tactical and small-sided games. Lower limb power (CMJ) and aerobic fitness (Yo-Yo intermittent recovery test level 1-IR1) were evaluated before and after the four-week training program. Yo-Yo IR1 performance was improved in both groups, but the improvement was greater for the HIIT_Train_ than Ball_Train_ group (468 ± 180 vs. 183 ± 177 m, *p* = 0.07). CMJ showed a non-significant improvement in the Ball_Train_ group (5.8 ± 8.8%, *p* = 0.16), but it decreased by 8.1 ± 9% (*p* = 0.001), in the HIIT_Train_ group. In conclusion, we have shown that a short pre-season period of training results in improvements in aerobic fitness in both groups, with high-intensity interval training showing superior adaptations than training with the ball. However, CMJ performance was reduced in this group, possibly suggesting higher fatigue levels and overload, and/or showing the effects of concurrent HIIT_Train_ and strength training in soccer.

## 1. Introduction

Pre-season is an important period of the year for setting the base for players’ performance during the competitive season, and its duration ranges from 4 to 12 weeks depending on the sport, athlete’s level, and the competitive season´s duration [1,2]. Training programs during this period are characterized by a higher volume and intensity compared to in-season and may also contain one or two friendly games per week [3,4]. The main aim of the pre-season is to improve all aspects of physical fitness (i.e., aerobic–anaerobic performance, and strength and power capacities) [5,6,7].

The ability to perform repeated bouts of intense exercise is an important characteristic of soccer players [8,9]. To improve repeated high-intensity running performance, coaches employ different training programs during the pre-season and the in-season periods. These programs are characterized by different volumes, bout durations and recovery intervals, such as high-volume moderate-intensity training [10], high-intensity interval training [HIIT] with and without the ball (with bouts lasting from 10 s to 6 min) [11], repeated sprint ability (RSA) training [12], or small-sided games (SSG) with different rules, number of players, and pitch dimensions [13,14].

Lower limb muscle power is also important for soccer, not only to improve performance but also to decrease the injury rate [5,6,7]. Resistance training using strength and power exercises with different loads, ranging from body weight to heavy loads, is commonly employed to increase lower limb muscle power in soccer players [5,11]. However, in soccer practice strength and endurance training are frequently performed in the same session [15,16] and this “concurrent training” has been shown to interfere with adaptations which promote muscle mass and power gain [17]. In terms of loading, some fitness trainers mainly use exercises against body mass only or light weights and devote more time to training with the ball, especially in teams with younger players [18], while others use heavier loads and basic weightlifting exercises, such as the half-squat, clean, etc. [5,19]. These different approaches of soccer coaches regarding the use of heavy or light strength power training in combination with high-intensity training with or without the ball in youth teams warrant further investigation. Thus, the aim of the present study was to compare two different concurrent training intervention programs, one based more on training with the ball (Ball_Train_) and strength training using plyometrics and exercises with body weight, while the other included sessions of strength training with moderate to high resistance and high-intensity running interval training without the ball (HIIT_Train_). Two different teams of elite youth players were measured before and after a 4-week pre-season period. It was hypothesized that both groups would improve their physical fitness parameters, with the HIIT_Train_ program showing a better improvement in their aerobic fitness and the Ball_Train_ group improving more in terms of lower limb power.

## 2. Materials and Methods

### 2.1. Experimental Approach

Greek soccer players from two youth elite teams (Superleague U19) participated in the present study and formed two groups. One group performed a higher percentage of aerobic training with ball and strength training using plyometrics and exercises with body weight (Ball_Train_), and the other group trained with high-intensity interval training without a ball (HIIT_Train_) and performed resistance training with weights in the same session. The experimental period lasted four weeks, and the evaluations were performed one week before and after the pre-season period. All participants were familiar with the tests used (i.e., CMJ and Yo-Yo intermittent recovery test level 1) as they had been evaluated regularly during the previous years. At the end of the intervention program, players rested for two days before post-testing in order to avoid any effect from acute or residual fatigue. Goalkeepers, and those who abstained from more than four training sessions (>15%) due to micro-injuries, were excluded from the study. Both teams trained for strength (two times/week), aerobic–anaerobic fitness without the ball, passing games, and tactical and small-sided games. However, Ball_Train_ performed a higher percentage of aerobic training with the ball and strength training using plyometrics and exercises with body weight, while the HIIT_Train_ group trained with HIIT without a ball and resistance training in the same session.

### 2.2. Subjects

Players from two youth teams (Superleague U19; 1st Division of the Greek league) participated in the present study. The first football team had 19 footballers on its roster and the second team had 23 footballers. Goalkeepers, and those who abstained from more than four training sessions (>15%) due to micro-injuries, were excluded from the study. The number of excluded players was 35% and 25% of the roster in each team. Thus, a total of 29 players were finally included in the study. Of those, 12 players of one team were allocated to the Ball_Train_ group (age: 17.8 ± 0.4 years, body mass: 73.9 ± 7.6 kg, height: 178 ± 0.1 cm, body mass index: 23.4 ± 2.2, and body fat: 9.6 ± 5.3%), and 17 players of the other team were allocated to the HIIT_Train_ group (age: 17.8 ± 0.7 years, body mass: 73.3 ± 5.0 kg, height: 179 ± 0.1 cm, body mass index: 22.8 ± 1.4, and body fat: 8.0 ± 2.3%). None of the players received any medication or illegal nutritional supplements, and they signed a written informed consent before entering into the research procedure. All procedures were in accordance with the Declaration of Helsinki and approved by the Ethics Committee of the School of P.E. and Sport Science, National and Kapodistrian University of Athens, Greece (Ref. number: 1048/2018).

### 2.3. Procedures

Vertical jump test: The countermovement jump (CMJ) was used to evaluate the vertical jump ability and lower limb power. Athletes were required to perform a downward movement followed by a complete, rapid extension of the lower limbs. The depth of the countermovement was self-selected to avoid changes in jumping coordination. The hands were placed on the hips throughout the whole movement and athletes were directed to jump as high as possible and land close to the take-off point with the same body posture as that at takeoff. They executed three maximal trials with a 1 min rest. The CMJ was performed on an electronic mat (CHRONOJUMP—Bosco system, Din-A4 297 × 210 m, Spain), and jump height was calculated using the following equation: h = t^2^ ∙ g ∙ 8^−1^. The highest jump was kept for analysis.

Aerobic fitness: Aerobic fitness was evaluated by the Yo-Yo intermittent recovery test level 1. The test consisted of repeated 2 × 20 m runs back and forth, with progressively increasing speed controlled by audio beeps from a CD in a computer with speakers (Bangsbosport.com). Between each running bout, the subjects had a 10 s active rest period, consisting of 2 × 5 m of jogging. When the subjects failed twice to reach the finishing lines, the distance covered was recorded and kept as the test result. $\dot{V}$O2max was calculated from the following equation: y = 0.0084 x + 36.4, where x is distance covered in the test and y is $\dot{V}$O2max in ml/kg/min [20]. The speed corresponding to $\dot{V}$O2max (v$\dot{V}$O2max) was estimated from the equation $\dot{V}$O2 (ml/kg/min) = 2.209 + 3.163 x speed (km·h^−1^). Heart rate was recorded for each player every 5 s using a wireless heart rate monitor worn around the chest (Suunto Team POD, Dual Comfort Belt, Finland).

Training Protocols: The Ball_Train_ program included six training sessions in the first week, and five training sessions plus a friendly game in the second and third week (Table 1). These sessions were held in the afternoon and contained exercises with the ball, such as passing games, small-side games, and tactical games, which aimed to develop the players’ ability to quickly transition from defensive to attacking positions and vice versa and to improve aerobic fitness of the players. The fourth week included four sessions in the afternoon similar to those in the previous weeks, and one session in the morning involving strength and interval endurance training. From the second week onwards, players performed a power strength session twice per week, in the form of circuit training, which involved 2 sets of 6 exercises (sumo squat, push-ups, 2-side hip raises, Russian twists, mountain climbers) with 30 s work and 30 s rest, and 3 sets of 12 repetitions of plyometric exercises, such as a unilateral jump on an unstable surface, horizontal jumps, and unilateral and bilateral jumps on hurdles with a height of 30 cm (Table 2), Each session lasted 24 min. Immediately after the strength training session, players performed endurance training, which, initially (starting at the second week) involved continuous running at a speed corresponding to 80% of the individual v$\dot{V}$O2max and, thereafter (weeks 3 and 4), included combinations of continuous and interval running at faster speeds (corresponding to 85–90% of the individual v$\dot{V}$O2max, see Table 2).

The HIIT_Train_ training program included six training sessions in the first week, and five training sessions plus a friendly game in the second and third week (Table 1). These sessions were held in the afternoon and contained passing games, small-side games, and tactical games. Furthermore, the weekly schedule of weeks two and three included two power strength sessions in the morning, where resistance training with free weights was employed (bench press, half squat, clean, hip-trust, leg curl, and leg extension, see Table 1 and Table 2). The fourth week included four afternoon sessions and two morning power strength and high-intensity interval training sessions. Resistance training was performed against loads based on the maximum strength (1RM) of each player. In the first session, a load of 60% 1RM was used, and players performed 3 sets of 10 repetitions in each exercise. In the following sessions, the intensity was gradually increased by 5% each week (i.e., from 75 to 95% 1RM) and the repetitions were decreased by two each week (i.e., from 10 to 2 repetitions), with the number of sets remaining unchanged (i.e., 3 sets) The recovery interval between sets was 2 min. Immediately after the resistance training, players performed high-intensity interval running training starting on the second week. Five sets of running (continuous and interval) were performed in week 1, at a running speed corresponding to 85% of the individual v$\dot{V}$O2max, with a 3 min recovery period between sets (Table 2). In weeks three and four, the number of sets was increased from five to six, and the running speed was also increased from 85% to 90% and finally to 95% of the individual v$\dot{V}$O2max, with a 3 min rest in between. From the second week onwards, there was one friendly game per week for each team (Table 1).

### 2.4. Statistical Analysis

Data are presented as means ± standard deviations (SD). Statistical analysis was performed using a statistical software (SPSS, Version 26, Chicago USA). A mixed-model two-way analysis of variance (two-way ANOVA) with repeated measures on one factor (pre- and post-training) and two groups (Ball_Train_ and HIIT_Train_) was used to examine differences in Yo-Yo IR1 performance (distance, VO2max, vVO2max) and CMJ performance. When a significant interaction was obtained, a Tukey’s post-hoc test was performed (unequal N HSD). The level of significance was set at *p* < 0.05.

## 3. Results

According to the design of the study, there were significant differences in the content of training between the two groups (Table 3). Specifically, the time of warm-up, strength training, number of sessions (*p* < 0.05), and interval training (*p* < 0.01) were significantly higher in HIIT_Train_ than Ball_Train_. Passing game time was higher in the Ball_Train_ group. No significant differences were found for tactical small-sided games, friendly matches, and total time between the two groups.

**Figure 1:**
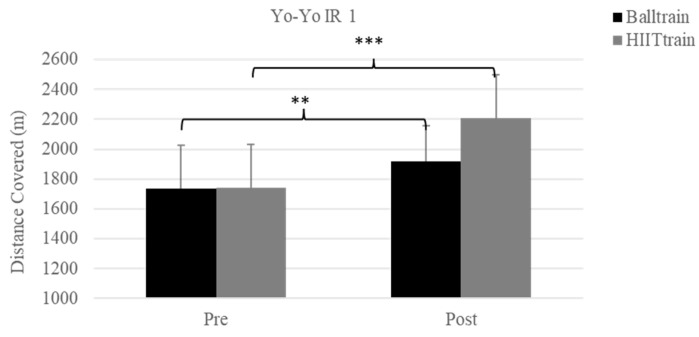
Yo-Yo intermittent recovery test level 1 (Yo-Yo IR 1) before (pre) and after training (post) in Ball_Train_ (black column) and HIIT_Train_ (gray column) groups. *** Statistical significantly difference between pre and post in HIIT_Train_ (*p* < 0.001) and ** Statistical significantly difference between pre and post in Ball_Train_ (*p* < 0.01).

**Figure 2:**
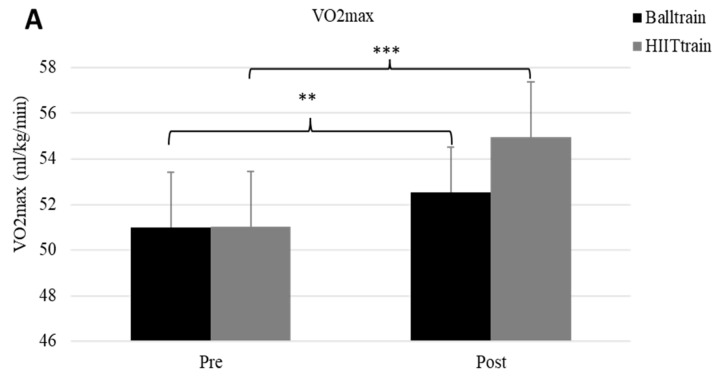

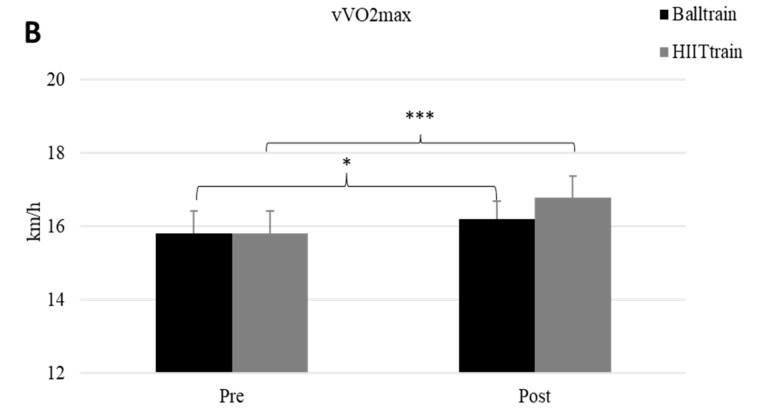
Maximum oxygen uptake (VO2max, panel (**A**)) before (pre) and after training (post) in Ball_Train_ (black column) and HIIT_Train_ (gray column) groups. ***: *p* < 0.001 between pre and post in HIITT_rain_ and **: *p* < 0.01 between pre and post in Ball_Train_; velocity at maximum oxygen uptake (vVO2max, panel (**B**)) pre and post in Ball_Train_ (black column) and HIIT_Train_ (gray column) groups. ***: *p* < 0.001 between pre and post in HIIT_Train_ and *: *p* < 0.05 between pre and post in Ball_Train_.

Maximum heart rate was similar before and after training for the two groups (before training: 195 ± 8 vs. 200 ± 8 bpm; after training: 196 ± 6 vs. 201 ± 7 bpm, for HIIT_Train_ and Ball_Train_, respectively). Aerobic fitness parameters (distance covered, vVO2max, and VO2max) were similar at pre-training in the two groups (1737 ± 290 vs. 1741 ± 291 m; 15.82 ± 0.60 vs. 15.83 ± 0.60 km/h; 50.99 vs. 51.03 ± 2.44 mL/kg/min, Figure 1 and Figure 2). Yo-Yo IR1 performance was improved in both groups, and the post-hoc test showed a tendency for a greater improvement in HIIT_Train_ than Ball_Train_ (2209 ± 288 vs. 1920 ± 238 m, *p* = 0.07). However, when the changes in the Yo-Yo IR1 test were compared between the two groups (i.e., post-training minus pre-training values), the improvement was significantly greater for the HIIT_Train_ compared with the Ball_Train_ group (468 ± 180 vs. 183 ± 177 m, *p* = 0.001).

CMJ was similar in the two groups before training (32.5 ± 3.6 and 37.4 ± 5.3 cm for HIIT_Train_ and Ball_Train_, respectively), and showed a non-significant improvement in the Ball_Train_ group (5.8 ± 8.8%, *p* = 0.16). However, in the HIIT_Train_ group it decreased (8.1 ± 9%, *p* = 0.001).

## 4. Discussion

The main aims of pre-season in soccer are to improve physical performance and to prepare the player for the competitive period using technical/tactical exercises aiming to enhance aerobic capacity with and without a ball [11,15,21], as well as agility and strength [7,15,19]. At the same time, by achieving the aforementioned aims, the injury risk during the in-season period is reduced [22]. Due to the relatively high training volume of endurance and strength training, the pre-season period may induce more fatigue when compared to the in-season period [3]. In the current study, the two groups used two different concurrent training programs, one based on strength with body weight and plyometric exercises in combination with passing and tactical games (Ball_Train_), while the other was trained with resistance and high-intensity interval training (HIIT_Train_). The main findings were that Yo-Yo IR1 performance was improved in both groups, with a greater improvement in HIIT_Train_ than Ball_Train_. Interestingly CMJ remained unchanged in the Ball_Train_ group but decreased in the HIIT_Train_ group.

One important finding of the present study was that the distance covered in Yo-Yo IR 1 showed a significant improvement (*p* < 0.001) in both groups, with HIIT_Train_ improving more than Ball_Train_. The results are in general agreement with previous studies, showing that pre-season training significantly improves aerobic fitness. According to their aerobic capacity, players in both groups may be classified as moderately trained at baseline [20,23,24]. However, the greater improvement in the HIIT_Train_ group compared to Ball_Train_ suggests that high-intensity training without the ball may ensure greater improvements in aerobic fitness, possibly due to the fact that all players train at high intensity for the allocated time, in contrast with training with the ball, during which a player may not train at high intensity for the entire duration of each bout [25]. Thus, despite the short duration of the intervention program (only 4 weeks), high-intensity interval training was more effective for inducing large improvements in aerobic fitness [8].

Despite the significant volume of strength and power training performed by both groups, CMJ performance showed no significant improvements in either group, with only a tendency of an increase in the Ball_Train_ group who used plyometric and body weight exercises. In contrast, CMJ performance decreased significantly in the HIIT_Train_ group, despite the high-load resistance training, which has been previously shown to improve lower limb explosive performance [5,15]. One possible explanation for this finding may be a residual neuromuscular fatigue caused by high volumes of endurance and high-load resistance training which may last more than 48 h, causing muscle soreness and reduced performance [19,26]. The combined load of high-intensity interval training that followed the strength with resistance training may induce muscle damage and inflammation lasting for several days [17,27]. Thus, a period of reduced training may be necessary for the benefits of this training to emerge [28] in order to prevent possible chronic fatigue, as may be the case for the HIIT_Train_ group [29]. On the other hand, circuit and plyometric training against the players’ body weight resulted in a slight, although non-significant, improvement of CMJ height during this 4-week pre-season period.

Another explanation for the reduced CMJ performance in HIIT_Train_ may be the combined effect of high-intensity aerobic training with high-load strength power training, known as the concurrent training effect on explosive performance, especially when aerobic and strength training are performed within the same training session [30,31]. The mechanisms of the reduction in strength and power following concurrent training in the same session involve both molecular and neural components [17]. In the present study, the reduction in the CMJ, when a combination of strength training and high-intensity aerobic training was used in the same session, was in agreement with the findings of Spiliopoulou et.al [17], who showed unchanged CMJ performance following training which combined strength and high-intensity interval aerobic training on a stationary bicycle for 6 weeks.

The present study has some limitations. The participants were members of two soccer teams, on whom we applied the training program. The number of participants was different in each team, due to the fact that 25–35% of each roster lost more than four sessions, and this was an exclusion criterion defined a priori. Due to technical and logistic issues, we could not obtain a rating of perceived exertion or global positioning system data, which would have provided further details regarding the load of the training. Furthermore, we could not obtain measurements of strength, as the coaches did not want their players to be tested maximally using weights. Finally, due to the fact that the training content differed in the type and duration of interval and strength training and passing games, the differences between groups are due to a combination of factors and not only the concurrent nature of the programs.

In conclusion, the current study showed that a short pre-season training period using intense sessions of aerobic training, either with or without the ball, (i.e., Ball_Train_ and HIIT_Train_) may significantly improve aerobic fitness in elite young soccer players. However, HIIT_Train_ was more effective in improving Yo-Yo IR 1 test performance. In contrast, CMJ performance was reduced in this group, possibly suggesting higher levels of chronic fatigue and overload and/or a negative effect of concurrent high-intensity aerobic training and high resistance strength training in these players. Thus, sport practitioners may be advised to provide adequate recovery when the training load is high during the pre-season, and to use concurrent training involving high-intensity aerobic and high-load strength training with caution, e.g., by separating them by a few hours or days, to avoid the negative effects of interference phenomenon on muscle power in young soccer players.

## Figures and Tables

**Table 1:** Pre-season training plan for the Ball_Train_ and the HIIT_Train_ group per week.

Ball_Train_							
First week							
Day	1	2	3	4	5	6	7
**PM**	Tec-tact training	Tec-tact training	Tec-tact training	Tec-tact training	Tec-tact training	Tec-tact training	Off
**Second and third week**							
**Day**	**1**	**2**	**3**	**4**	**5**	**6**	**7**
**PM**	Tec-tact training	Power strength, tec-tact training	Interval, tec-tact training	Power strength, tec-tact training	Tec-tact training	Friendly game	Off
**Fourth week**							
**Day**	**1**	**2**	**3**	**4**	**5**	**6**	**7**
**AM**	X	X	X	X	Power strength, interval training	Off	Off
**PM**	Power strength, tec-tact training	Tec-tact training	Friendly game	Tec-tact training	Tec-tact training		
**HIIT_Train_**							
**First week**							
**Day**	**1**	**2**	**3**	**4**	**5**	**6**	**7**
**PM**	Tec-tact training	Tec-tact training	Tec-tact training	Tec-tact training	Tec-tact training	Tec-tact training	Off
**Second and third week**							
**Day**	**1**	**2**	**3**	**4**	**5**	**6**	**7**
**AM**	X	Power strength, interval training	X	Power strength, Interval Training	X	X	Off
**PM**	Tec-tact training	Tec-tact training	Tec-tact training	Tec-tact training	Tec-tact training	Friendly game	
**Fourth week**							
**Day**	**1**	**2**	**3**	**4**	**5**	**6**	**7**
**AM**	Power strength, interval training	X	X	X	Power strength, interval training	Off	Off
**PM**	Tec-tact training	Tec-tact training	Friendly game	Tec-tact training	Tec-tact training		

Ball_Train_, group training with the ball; HIIT_Train_, group training with strength + high intensity interval training; tec-tact, technical and tactical training (includes small-sided games and passing games); AM, sessions in the morning; PM, sessions in the afternoon.

**Table 2:** Strength and interval weekly training programs for the two groups.

Group Ball Training + Body Weight-Plyometric Training (Ball_Train_)						
Type	Circuit	Plyometric	Interval Training			
			First Week	Second Week	Third Week	Fourth Week
**Exercises**	Sumo squat Push-ups Hip extension Russian twist Mountain-climbers	Unilateral jump on an unstable surface Horizontal jump Unilateral and bilateral vertical jump (30 cm hurdles)	-	Continuous running	One set of continuous running, one set of 50 m running–50 m jogging, one set of 10 s–10 s/work–rest	Two sets of continuous running, two sets of 50 m running–50 m jogging, Two sets of 10 s–10 s/work–rest
**Sets**	2	3	-	1	3	6
**Reps/Duration**	30 s	12 reps	-	6 min	6 min	4 min
**Intensity**	Low	Moderate–high	-	80% v$\dot{V}$O2max	85% v$\dot{V}$O2max	90% v$\dot{V}$O2max
**Rest**	30 s	1 min	-	-	3 min	3 min
**Group High-intensity Interval + Resistance Training (HIITTrain)**						
**Type**	**Resistance**		**Interval Training**			
			**First week**	**Second week**	**Third week**	**Fourth week**
**Exercises**	Clean Bench press Half-squat Hip-trust Leg curl Leg extension		-	Two sets of continuous running, two sets of 50 m running–50 m jogging, one set of 10 s–10 s work–rest	Two sets of continuous running, two sets of 50 m running–50 m jogging, two sets of 10 s–10 s work–rest	Two sets of continuous running, two sets of 50 m running–50 m jogging, two sets of 10 s–10 s work–rest
**Sets**	3		-	5	6	6
**Reps/Duration**	2–10 reps		-	6 min	5 min	4 min
**Intensity**	Moderate–high		-	85% v$\dot{V}$O2max	90% v$\dot{V}$O2max	95% v$\dot{V}$O2max
**Rest**	2 min		-	3 min	3 min	3 min

Abbreviations are as follows: reps, repetitions; VO2_max_, maximal oxygen consumption; vVO2_max_, velocity at maximal oxygen consumption.

**Table 3:** Time devoted to each part of the training session for the Ball_Train_ and the HIIT_Train_ group. Values are mean ± standard deviation (SD).

		Ball_Train_		HIIT_Train_		
Training Session Part	Time	Total in 4 Weeks	Total Per Week	Total in 4 Weeks	Total Per Week	*p* Value
Warm-up	(min)	268	67.0 ± 7.0	337	84.3 ± 7.8	0.017
Interval training	(min)	48	16.0 ± 9.2	168	56.0 ± 6.9	0.005
Tactical small-sided games	(min)	706	176.5 ± 24.8	648	162.0 ± 31.0	0.529
Passing game	(min)	350	87.5 ± 26.2	162	40.5 ± 15.6	0.027
Strength training	(min)	96	24 ± 0.0	396	99.0 ± 42.0	0.038
Friendly match	(min)	270	90	270	90	1.000
Cooldown	(min)	220	55.0 ± 5.8	260	65.0 ± 5.8	0.050
Total Time	(min)	1958	489.5 ± 123.9	2241	560.3 ± 94.5	0.242
Number of sessions		22	5.5 ± 0.6	26	6.5 ± 0.6	0.050

Ball_Train_, group training with the ball; HIIT_Train_, group training with high-intensity interval + strength training in the same session twice per week; Total, sum time in 4 weeks expressed in minutes; Total per week, expressed as mean± standard deviation.
